# Generating Consensus on Good Practices in the Care of Portuguese Internal Medicine Patients Facing Imminent Death: A Delphi Study

**DOI:** 10.3390/healthcare12191990

**Published:** 2024-10-05

**Authors:** Rui Carneiro, Manuel Luís Capelas, Catarina Simões, Elga Freire, António Henriques Carneiro

**Affiliations:** 1 Palliative Care Team, Internal Medicine, Emergency and Intensive Care Department, Hospital da Luz—Arrábida, 4400-346 Vila Nova de Gaia, Portugal; catarina.simoes@santamariasaude.pt; 2 Faculty of Health Sciences and Nursing, Universidade Católica Portuguesa, 1649-023 Lisbon, Portugal; luis.capelas@ucp.pt; 3 Portuguese Observatoryof Palliative Care, Universidade Católica Portuguesa, 1649-023 Lisbon, Portugal; 4 Centre for Interdisciplinary Health Research, Universidade Católica Portuguesa, 1649-023 Lisbon, Portugal; 5 Nursing Department, Escola Superior de Saúde de Santa Maria, 4049-024 Porto, Portugal; 6 Internal Medicine and Palliative Care Team, Centro Hospitalar e Universitário de Santo António, 4099-001 Porto, Portugal; elgafreire@sapo.pt; 7 Internal Medicine, Emergency and Intensive Care Department, Hospital da Luz—Arrábida, 4400-346 Vila Nova de Gaia, Portugal; antonio.henriques.carneiro@hospitaldaluz.pt

**Keywords:** terminal care, palliative care, organization, quality of life, Delphi method

## Abstract

Context: Modern medicine aims to ensure a world in which all people experience a good end of life as an integral part of their life journey. A good end-of-life experience means dying with dignity and receiving the best healthcare based on scientific evidence. Objective: This study aims to reach a consensus about the contents of a comprehensive instrument based on the 10/40 Model of the International Collaborative for the Best Care for the Dying Person for evaluating inpatients facing imminent death in Portuguese internal medicine wards and a proposal for anticipatory medication for symptom control in inpatient and home care settings. Methods: We employed the Delphi method and conducted various rounds of questionnaire administration to 23 Portuguese internists competent in palliative medicine. Data were obtained in July and September of 2022. Results: Consensus was reached among the expert panel on the diagnostic, initial assessment, monitoring, and after-death care items of the tool, with minor adjustments to wording or content. However, it was not possible to reach a consensus on most of the proposals presented for anticipatory medication for symptomatic control. Conclusion: We present the consensus about the contents of a comprehensive instrument for evaluating inpatients facing imminent death in Portuguese internal medicine wards. Best practices in this setting were defined from the point of view of internists with expertise in palliative care. However, the best pharmacological practices still require further reviews of the literature and consensus.

## 1. Introduction

In Portugal, two-thirds of deaths occur in hospitals [1], and this number is expected to increase significantly in the coming decades [2]. Internists have a broad presence in hospital wards and are trained in addressing patients in a holistic and ethical manner, considering all psychological and medical factors to improve their quality of life and provide palliative care at the end of life [3]. The prevalence of terminal illnesses in patients in internal medicine wards is around 20 to 25%, and the majority of such illnesses are non-oncological [4,5]. A study conducted in 2008 in a department of internal medicine showed that 75% of nurses and 50% of doctors considered that the death of their patients involved considerable and unnecessary suffering [6]. More than a decade later, these numbers only decreased to 43% and 29%, respectively, and most professionals still lack any training in the field of palliative care [7]. The situation of the last hours or days of life, known as “imminent death syndrome”, is the final stage of the end-of-life journey [8]. It is characterized by global, irreversible bodily dysfunction and presents its own set of symptoms [8,9], meaning that the needs of patients and their families change drastically. Properly organizing care at this stage facilitates peaceful deaths and fosters healthy grieving [10,11].

The current concept of good end-of-life care includes dying without pain, being cared for with dignity, and choosing (if possible) place of care, which should be supported by the best available scientific evidence [12,13]. The national literature on care in the last hours or days of life shows that, in small series, the application of a model focused only on the acute disease in patients who are dying leads to suboptimal performance [14,15]. Such models mainly focus on needs assessments and symptomatic control, with little evidence on the typology of non-pharmacological treatments or about addressing the cultural and social needs of patients and their families [14,15].

In 2021, the publication of the “Guia de consenso sobre boas práticas nos cuidados de fim de vida” (“Consensus Guide on Best Practices in End-of-Life Care”) by the Spanish and Portuguese Societies of Internal Medicine represented a significant advance in this specialized field and for patients, providing general guidelines about the care of patients in their last hours or days of life. The guide included the identification of imminent death syndrome, the stratification of patients’ needs, and the development of an individualized care plan [16]. However, the guide did not fully articulate how imminent death care should be organized in practice, and there is no agreed-upon instrument for supporting national health teams in such a task [17]. Concurrently, there is no training model that provides internists with practical competence in accompanying patients and their families in their last hours or days of life, which can contribute to mismatches in the therapeutic plan and poor communication, making it difficult to audit and benchmark good practices.

The International Collaborative for the Best for the Dying Person is a global consortium of clinicians and researchers—from 22 countries across all continents—that aims to ensure a world in which all people experience a good end of life as an integral part of their life journey [18]. They support the production and dissemination of knowledge based on a solid research methodology. One of their main developments is the 10/40 Model, a synthesis of best care practices based on the best available scientific evidence, with 10 care principles and 40 expected outcomes [12]. The 10/40 Model is derived from an extensive review of the evidence under the auspices of OPCARE9, with advanced consensus obtained on optimal care for these patients across themes such as semiotics of approaching death, end-of-life decisions, comfort care, and psycho-social support [18,19]. These elements were incorporated into the model of documentation and care delivery, and its principles have been used in several countries to guide their end-of-life policies and to facilitate consistent documentation of care in accordance with the best scientific evidence [20]. The 10/40 Model allows any service, institution, region, or country to develop and implement a methodology for caring for patients in the last hours or days of life and to promote equitable access to excellent care in the final days of life, even in the absence of specialized palliative care access [20]. In Portuguese internal medicine, it is crucial to have such a shared and adapted framework for evaluating and monitoring the comfort of dying patients.

## 2. Aim

The aim of this study is to assess the content validation via consensus among experts of a comprehensive instrument for evaluating inpatients facing imminent death in Portuguese internal medicine wards.

## 3. Methods

### 3.1. The MiMI Project

The MiMI Project (Morte iminente em Medicina Interna; Imminent Death in Internal Medicine), ongoing since 2022, is a collaborative effort promoted by the Portuguese Society of Internal Medicine and the International Collaborative for the Best Care for the Dying Person, with the support of the Faculty of Health Sciences and Nursing of the Universidade Católica Portuguesa [21]. The aim of the MiMI Project is to fill an important knowledge gap regarding the care of people in their last hours or days of life and to provide a basis for improving their quality of life. The MiMI Project is being developed in terms of five main axes [22]: (I) the national standardization of good practice documentation to guide the evaluation and monitoring of patients facing imminent death; (II) a prospective audit of the care for internal medicine inpatients facing imminent death; (III) the characterization of the experiences of internists while caring for patients in dying situations; (IV) the identification of training needs in end-of-life care; and (V) the development of a curriculum, a pedagogical model, and a training program in the relevant areas. This study focuses on axis (I) of this project.

### 3.2. Study Design

A semi-structured Delphi questionnaire was administered to an expert panel of physicians with specialization in internal medicine and competence in palliative medicine to assess their level of agreement/disagreement with the elements of care included in the documentation instrument. We used the Delphi method to systematically generate consensus by using panels of carefully selected experts. Consensus generated by expert opinion contributes to solutions for real-world problems with direct and practical results [23], as intended in this research, especially because it spans an area with scientific and, at times, empirically based knowledge limitations.

The number of questionnaire rounds was not defined in advance and was determined according to the generated consensus. After each round of questionnaires, an informative report of the obtained consensus and aggregated comments was provided before proceeding to the next round.

We followed the methodology proposed by the “Guidance on Conducting and Reporting Delphi Studies in Palliative Care” (CREDES) to ensure the methodological quality of the presented results [24].

Data collection took place in July and September of 2022.

#### 3.2.1. Expert Panel and Recruitment

Using the convenience sampling method, specialists in internal medicine competent in palliative medicine were recruited via direct invitation to participate in this study, sent by email from the Portuguese Society of Internal Medicine. We reviewed all working palliative care teams on the mainland and islands listed in the Portuguese National Health Directory [25,26,27]. Participants operating within hospital-based palliative care teams (intra-hospital support teams and inpatient units) were contacted (by phone, email, and social media) and asked to refer and invite other physicians meeting the inclusion criteria. Out of the 60 listed palliative care teams, we were unable to contact 4 teams, and 16 did not fulfill the inclusion criteria. Other professionals were identified through a snowball strategy, using the direct contacts of the researchers. According to the Portuguese Medical Association, 52 physicians in Portugal have characteristics compatible with the inclusion criteria. We successfully contacted 36 physicians (67% of the national database of internists competent in palliative medicine), and only 1 professional declined to participate.

All participants filled out an informed consent form. Confidentiality of the collected data was ensured through a coding system that prevented participant identification, even for the research team.

#### 3.2.2. Survey Instrument

The survey instrument presented to the panelists was prepared in two parts. The first component relates to the organizational structure of care, based on the principles of the 10/40 Model from the International Collaborative for the Best for the Dying Person [20]. This clinical tool had been previously adapted to Portuguese and assessed for formal congruence by this international committee. This clinical tool includes elements related to (1) diagnosis of the last hours or days of life; (2) assessments of patient and family communication skills; (3) assessments of the physical, psychological, and spiritual needs of patients and families; (4) the monitoring and documentation of the presence of main symptoms (pain, dyspnea, noisy breathing, restlessness, nausea/vomiting, and fever); (5) the verification of an adequate prescription for the control of main symptoms; (6) interventions to be suspended or minimized; (7) interventions to be implemented or emphasized; (8) post-mortem care; and (9) a review of the diagnosis of the last hours or days of life situation. The second component of the instrument deals with the structure of anticipatory pharmacological care for the cardinal symptoms that may arise during an imminent death situation. A proposal for pharmacological use in the hospital and at home, in case of a fast-track discharge for home death, was presented to the experts, symptom by symptom. The therapeutic proposals were based on the available scientific literature [28]. In the absence of robust evidence regarding the anticipatory prescription of medication in case of hospital discharge for home death and recognizing the time limitations for caregiver training in the use of subcutaneous routes (preferential route in these conditions) and for the activation of palliative care resources, the authors developed a set of prescription proposals using active principles suitable for administration in anatomical cavities (oral and/or rectal) and according to current clinical experience.

#### 3.2.3. Data Collection Methods

Two online questionnaires were created using Microsoft Forms and sent via email to the participants. The participants submitted their answers through a secure link. The two questionnaires detailed the following: (1) professional characteristics (years of professional experience) and educational background (year of certification in palliative medicine); (2) agreement with the items in the instrument using a Likert scale: 1—disagree, 2—partially disagree, 3—neither agree nor disagree, 4—partially agree, and 5—fully agree. Participants also had the opportunity to add free comments.

#### 3.2.4. Data Analysis

For each topic of the instrument, the degree of agreement was characterized based on the frequency of responses, median, and interquartile range, categorized as follows: very high (≥80% agreement; median 5 and interquartile range 0); high (≥80% agreement; median 4–5 and interquartile range 1); moderate (60–79% agreement; median 4 or lower and interquartile range 1); and low (<60% agreement; median below 4 and interquartile range above 1).

Consensus among experts was reached when the level of agreement was “very high” or “high”. When consensus was not achieved, free comments were considered for rephrasing or changes to the content. In the absence of comments, the research team proceeded to the next questionnaire round to confirm the lack of consensus before removing the topic from the instrument.

#### 3.2.5. Ethical Considerations

This study obtained approval from the Ethics Commission for Health of the Universidade Católica Portuguesa (process number: 198).

## 4. Results

Out of the 35 professionals who agreed to participate in this study and provided informed consent, 28 responded to the questionnaire in the first round, and 23 (final response rate of 65.7%) responded in the second round. The results showed no difference in the years of professional experience between the two groups (Table 1).

### 4.1. Results of Round 1

Table 2 presents the results of the questionnaire rounds. All items representing objectives of care organization (except item 26: “Consider the use of saliva substitutes”) obtained a “high” or “very high” level of agreement, thus achieving consensus. Although consensus was reached for some items with “very high” or “high” agreement, there were comments suggesting minor phrasing changes (Table 3). Since there were only a fewcomments in this first round, the research team decided not to incorporate these suggestions in the document for consideration in the next round.

Regarding the proposed anticipatory medication prescriptions, only the items related to hospital-based therapy for pain (item 37), dyspnea (item 39), delirium (item 41), and fever (hospital and home settings: items 47 and 48) reached consensus. However, no comments were generated by respondents about the reasons for the lack of agreement on the remaining items. The research team decided to proceed with all items to the second round and emphasized the importance of providing reasons for disagreement.

### 4.2. Results of Round 2

Consensus was re-encountered for all items in the organizational component of care, with “very high” or “high” agreement with all care principles, except for item 26 (“Consider the use of saliva substitutes”), which obtained a high median agreement but with reasonable dispersion of opinions. This item was removed in the final version. Respondents considered that the most important aspect of oral care is humidification, using any available means (not necessarily using saliva substitutes), as already proposed in item 24 (“It is important to include the optimization of oral care in patients in their last days of life”), which reached consensus in both rounds (Table 2).

Comments were generated by respondents focused on phrasing issues in the questionnaire, the categorization of symptom assessment, and the source of information on advance directives (Table 3). The initial phrasing of item 1 was altered to remove the adverb “ethically”, where it was stated that the diagnosis of the last hours or days of life situation is “made in the presence of a set of signs and symptoms, and in the absence of (ethically) reversible etiology: prostration, loss of oral intake, death rattle, poor peripheral perfusion, and Cheyne–Stokes respiration.” All other comments, while relevant, did not raise serious doubts about the meaning and reading of the care objective. Therefore, the items were maintained according to the initial proposal (as long as consensus was reached).

Regarding the proposed pharmacological anticipatory therapy, consensus was reached for the hospital-based control of pain (item 37), dyspnea (item 39), restlessness (item 41), death rattle (item 45), and fever in hospital and home settings (items 47 and 48). All other proposals received “moderate agreement”, and items concerning home-based pharmacological control of pain (item 38), dyspnea (item 40), nausea/vomiting (item 44), and death rattle (item 46) received “low agreement”.

In this round, due to the research team’s emphasis on the invitation for feedback at the time of the second invitation to participate, the respondents provided multiple comments regarding the proposed pharmacological options (Table 3). For home-based pain and dyspnea control, issues were raised regarding the off-label use of oral transmucosal formulations of fentanyl in opioid-naive patients; the use of oral morphine formulations rectally was considered “undignified”, even if temporary; and the possibility of titrating transdermal opioids in situations of imminent death with prolonged duration. For agitation control, other pharmacological options or routes of administration were suggested, from exploring orodispersible or oral formulations (sublingual) of neuroleptics to the possibility of using lower doses than those recommended for midazolam. The respondents pointed out that the pharmacological proposal for home use depends greatly on the resources available in the community and, therefore, disagreed with the proposals presented here.

For the pharmacological control of nausea/vomiting, concerns were raised about the recommended doses of metoclopramide, and other possibilities, such as neuroleptics or corticosteroids with antiemetic purposes, were proposed. Regarding the pharmacological control of death rattle, the panel pointed out the lack of robust scientific evidence for the use of anticholinergics for symptom control. Some respondents mentioned that their use should be prophylactic rather than after the onset of symptoms or even for the use of different anticholinergics from those proposed (e.g., ipratropium) and through different routes than those proposed (e.g., rectal butylscopolamine). Questions were also raised about the dose of furosemide and whether higher bolus doses could cause skin discomfort. The lack of consensus and the comments demonstrate the diversity of practices, even in a palliative care environment, despite little robust scientific evidence regarding the effectiveness of the main pharmacological principles employed. No additional Delphi questionnaire rounds were conducted because the systematization or standardization of pharmacological care practices for home-based care in situations of imminent death, especially in the absence of palliative care resources, requires a different methodological approach, as discussed in the next section.

## 5. Discussion

This study intends to reach a consensus about the contents of a comprehensive instrument for evaluating inpatients facing imminent death in Portuguese internal medicine wards. By defining what constitutes best practices in this setting, this study constitutes the first attempt in our country to develop a shared and standardized practical checklist on what to address when making diagnoses and evaluating the physical and psycho-spiritual needs of patients and their relatives.

We found a high consistency between questionnaire rounds. Most of the consensus items had an agreement level near or above 90%, pointing toward a strong consensus being reached. This was supported by the free comments in round 2, where experts justified their sustained level of agreement.

The consensus was generated by a panel of Portuguese internal medicine doctors competent in palliative medicine. These professionals are aware of the strengths and limitations of working in internal medicine wards and were selected after contacting all eligible experts at the national level. They had substantial clinical experience and are highly differentiated in the field of study. Moreover, the participants were committed to this study, as shown by the low drop-out rate between rounds (18%).

This now-available instrument provides a systematized structure of care that is congruent with the Iberian consensus on best practices in end-of-life situations [16] and identifies the tangible principles and goals of care, making it unequivocally practical to use. Although it stems from the 10/40 Model of the International Collaborative for the Best for the Dying Person [20], the present instrument does not address how the multidisciplinary internal medicine team organizes and delivers care. However, despite not constituting a model of care [29], this clinical tool may still be used as a shared framework and may foster team discussion on clinical decisions.

The findings of our study align with the recently updated international consensus on the best practices in the care of patients who are dying included in the 10/40 Model, reaffirming the 10 key principles and 40 core outcomes as the state of the art in the care of patients who are dying [30]. Furthermore, our initial instrument was previously deemed congruent by the Coordinating Centre of the International Collaborative for the Best for the Dying Person, grounding the items used in our instrument in the established body of knowledge.

Recently, the National Hospice and Palliative Care Organization Standard of Practices reviewed the principles of care, including in the last days of life, which included ensuring patient comfort and family support and respecting cultural and spiritual needs, alongside good symptom management [31]. All these principles are included in our instrument. The items pertaining to inpatient pharmacological approaches to controlling the symptoms in this phase of life also aligned with the criteria denoted by recent guidelines, such as the 2023 Northern Ireland guidance on the management of symptoms in adults in their last days of life [32]. This agreement between the items and guideline criteria, alongside the expert panel’s refinement of the phrasing of some of the items, contributes to the content validity of our instrument.

The proposed instrument includes elements for systematically identifying patients’ needs in their last days of life, suggests paying close attention to the care setting to promote comfort, including routine symptom surveillance, and emphasizes the importance of addressing communication issues. This clinical tool also stresses the importance of adjusting the pharmacological and non-pharmacological care plans. Full implementation of this instrument must occur alongside educational opportunities to enhance healthcare professionals’ abilities in communication, decision-making, and teamwork. These aspects were contemplated within the framework of the MiMI Project.

Consensus could be reached for the pharmacological practices proposed for the symptomatic relief of patients facing imminent death admitted to internal medicine services, particularly those through the parenteral route, except for nausea/vomiting (due to concerns about the high proposed dosages and the possibility of other options) and respiratory secretions (due to the presence of equivocal scientific evidence). When addressing hospital discharge, specifically for home death, the low agreement values and their dispersion, along with the comments of the respondents, demonstrated quite diverse practices, even in a palliative care environment. Limited scientific evidence regarding the efficacy of the main pharmacological principles used by non-parenteral routes was stressed. The respondents were very clear in pointing out that the typology of pharmacological care depends greatly on the resources available in the community. Portugal still faces significant obstacles in providing comprehensive end-of-life care in a home setting, including a shortage of formal palliative care support in the community. Outside the palliative care setting and without specific training or clear guidelines, it may be challenging for the internal medicine team to schedule medication infusions and/or the administration of subcutaneous boluses (the challenges include drug availability, caregiver training, and supervision). This may be common in the UK, but evidence of such standard practices is scarce in other countries [33]. Although these anticipatory prescriptions are well accepted by the family and perceived by healthcare professionals as being useful in symptom control, a systematic review and narrative synthesis excluding non-injectable drugs found no robust evidence of clinical effectiveness [33]. The research team, therefore, decided to eliminate the entire pharmacological component of the instrument for end-of-life care and to change the research methodology for this topic. We also decided to undertake a systematic review of the literature on home-based pharmacological care in situations of imminent death, specifically exploring routes of administration that do not require caregiver training and, preferably, using drugs available in community pharmacies. After this systematic review, it will be appropriate to hold a consensus conference with internists with experience in palliative care on the organization of care in imminent death scenarios in hospital settings, especially during the transition to home care when no palliative care team is readily available.

There are limitations to the present study. Only medical professionals were included. Certainly, including other professionals in the internal medicine realm who are also involved in end-of-life care could have brought improvements to the instrument. However, we believe that this bias was reduced as we found a very high agreement with the 10/40 Model principles, which were developed with international and multiprofessional input [20]. As a means of reducing the selection bias of direct invitation, the attempt to contact all national palliative care hospital-based teams was considered an effective mitigating strategy.

The Delphi method is useful for conciliating existing knowledge with experience but not for solving problems or generating new solutions [34]. The consistent lack of agreement regarding the pharmacological approaches proposed for the transition to home care without continued palliative care supervision is an important problem for internal medicine teams, as well as a research priority, but no directions for future research may be drawn from the panelist opinions.

A further limitation is related to the quality of evidence and its generalization. Even when expert opinion is the mainstay of Delphi studies, it is still regarded as a poor basis for making judgments in healthcare [35]. Although the consensus around the items mirrors the standard of clinical care in current palliative care settings, the proposed approach may not serve well in internal medicine wards and may be perceived as infeasible by general internists lacking palliative care skills. Therefore, the presented framework should not be generalized without supervision. However, the framework may serve as a comprehensive starting point for supporting stakeholders (such as the Portuguese Internal Medicine Society) in developing a quality-of-care improvement methodology that validates competency in internal medicine teams and their specific skills, performance, and certification. The MiMI project intends to serve as leverage in such a path.

## 6. Conclusions

The results of this study provide Portuguese internal medicine teams with an instrument that can guide the evaluation of inpatients in their last hours or days of life. This study presents the consensus of experts on the first national practical checklist on how to address the diagnosis and evaluation of the physical and psycho-spiritual needs of patients and their relatives. Moreover, this clinical tool defines best practices in this setting from the perspective of internists with expertise in palliative care. Finally, this framework should not be generalized without supervision; however, it may serve as a comprehensive starting point for supporting stakeholders in developing a quality-of-care improvement methodology.

## Figures and Tables

**Table 1:** The Delphi method questionnaire results for each round according to participants’ characteristics.

	Round 1	Round 2
N	28	23
Clinical experience (years)		
Mean	21.6 *	22.3 *
Standard deviation	10.9	11.4
Year of Palliative Certification **		
2015	7	5
2016	3	1
2017	3	2
2018	0	0
2019	2	3
2020	8	7
2021	3	2
2022	2	2

* *t*(49) = −0.226, *p* = 0.839. ** “Competência em Medicina Paliativa”, from the Portuguese Medical Association; in Round 2 one respondent did not provide this information.

**Table 2:** The results from the Delphi method questionnaire rounds.

	Round	Median Agreement *	IQR **	Degree of Agreement	Consensus
Diagnóstico da Síndrome de Morte Iminente (situação de últimas horas ou dias de vida) *Diagnostic of Imminent Death Syndrome (last hours or days of life)*					
1. É feito na presença de conjunto de sinais e sintomas, na ausência de etiologia (eticamente) reversível: prostração, perda de via oral, estertor, má perfusão periférica, respiração de Cheyne–Stokes. *Diagnosis is made in the presence of a set of signs and symptoms and in the absence of an (ethically) reversible etiology: prostration, loss of oral intake, death rattle, poor peripheral perfusion, and Cheyne–Stokes breathing.*	**1**	5	1	96.4%	**Yes**
	**2**	5	1	95.7%	**Yes**
2. A decisão é clínica e implica avaliação médica obrigatória. *The decision is clinical and requires mandatory medical evaluation.*	**1**	5	0	100%	**Yes**
	**2**	5	0	95.7%	**Yes**
Avaliação das habilidades de comunicação do doente e da família *Evaluation of patient and family communication skills*					
3. Avaliar da possibilidade de comunicar com o doente/família. *Assess the possibility of communicating with the patient/family.*	**1**	5	0	96.4%	**Yes**
	**2**	5	0	95.7%	**Yes**
4. Identificar diretivas antecipadas de vontade do doente. *Identify the patient’s advance directives.*	**1**	5	1	85.7%	**Yes**
	**2**	5	0	95.7%	**Yes**
5. Avaliar vontade familiar em acompanhar em presença física permanente o doente. *Assess the family’s willingness to continue physically accompanying the patient.*	**2**	5	0	100%	**Yes**
	**2**	5	0	100%	**Yes**
6. Confirmar os contactos da família bem como a certificação dos horários de possibilidade de contacto. *Confirm the family contacts as well as their contact availability.*	**1**	5	0	96.4%	**Yes**
	**2**	5	0	100%	**Yes**
Avaliação de necessidades físicas, psíquicas e espirituais do doente e família *Assessment of physical, psychological, and spiritual needs of the patient and family*					
7. O doente é cuidado num ambiente físico apropriado à satisfação das suas necessidades individuais como, por exemplo, cortinas, telas, ambiente limpo, espaço suficiente na cabeceira, considerar fragrâncias, silêncio, música, luz, escuridão, quadros, fotografias, campainha. *The patient is cared for in a physical environment appropriate for meeting their individual needs, for example, the availability of curtains or screens, being in a clean environment, having sufficient space at the bedside, not using strong fragrances, resting in silence or listening to music, having appropriate levels of light and darkness, whether pictures or photographs are on display, and the availability of a call bell.*	**1**	5	0	100%	**Yes**
	**2**	5	1	95.7%	**Yes**
8. Avaliar as necessidade do doente ou família em rever aspetos dos cuidados de fim de vida cruciais ao sistema de crenças. *Assess the need for the patient or family to review any aspects of their end-of-life care that are crucial to their belief system.*	**1**	5	0	100%	**Yes**
	**2**	5	0	95.7%	**Yes**
9. Avaliar a necessidade de ativação de serviço de capelania/assistência espiritual. *Assess the need to activate chaplaincy/spiritual assistance services.*	**1**	5	0	100%	**Yes**
	**2**	5	0	100%	**Yes**
Vigilância e documentação da presença de 5 sintomas cardinais *Surveillance and documentation of the presence of 5 cardinal symptoms*					
10. Registar, pelo menos 1 vez por turno, a presença/ausência de dor. *Record, at least 1 time per shift, the presence/absence of pain.*	**1**	5	0	100%	**Yes**
	**2**	5	0	100%	**Yes**
11. Registar, de pelo menos 1 vez por turno a presença/ausência de dispneia. *Record, at least 1 time per shift, the presence/absence of dyspnea.*	**1**	5	0	100%	**Yes**
	**2**	5	0	100%	**Yes**
12. Registar, pelo menos 1 vez por turno, a presença/ausência de náusea/vómito. *Record, at least 1 time per shift, the presence/absence of nausea/vomiting.*	**1**	5	0	100%	**Yes**
	**2**	5	0	100%	**Yes**
13. Registar, pelo menos 1 vez por turno, a presença/ausência de agitação. *Record, at least 1 time per shift, the presence/absence of restlessness.*	**1**	5	0	100%	**Yes**
	**2**	5	0	100%	**Yes**
14. Registar, pelo menos 1 vez por turno, a presença/ausência de estertor. *Record, at least 1 time per shift, the presence/absence of a death rattle.*	**1**	5	0	100%	**Yes**
	**2**	5	0	95.7%	**Yes**
Verificação da existência de prescrição adequada para controlo dos 5 sintomas cardinais *Verification of the adequate prescription for controlling the 5 cardinal symptoms*					
15. Confirmar da existência de prescrição adequada para a eventualidade de descontrolo de sintomas: dor, dispneia, náuseas/vómitos, agitação e estertor. *Confirm the existence of an appropriate prescription for symptom control: pain, dyspnea, nausea/vomiting, restlessness, and death rattle.*	**1**	5	0	100%	**Yes**
	**2**	5	0	95.7%	**Yes**
16. Confirmar da existência de vias de administração de maior comodidade, incluindo alternativa em caso de perda de via oral e falência de acessos endovenosos. *Confirm the existence of more convenient administration routes, including alternatives in the case of loss of oral intake and failure of intravenous access.*	**1**	5	0	100%	**Yes**
	**2**	5	0	95.7%	**Yes**
Intervenções a suspender ou a minimizar importância *Interventions to be suspended or minimized*					
17. Confirmar informação sobre indicação para suporte vital ou para manobras de reanimação. *Confirm information on the indication for life support or resuscitation status.*	**1**	5	0	96.4%	**Yes**
	**2**	5	0	100%	**Yes**
18. Confirmar desativação de CDI (se existente). *Confirm the deactivation of ICD (if existing).*	**1**	5	0	89.3%	**Yes**
	**2**	5	0	100%	**Yes**
19. Descontinuar a avaliação rotineira de parâmetros vitais, vigiando sinais de febre e, na sua presença, documentar a temperatura corporal bem como descontinuar pesquisa de glicemia capilar. *Discontinue routine evaluation of vital parameters, monitor fever signs, and, in their presence, document body temperature and discontinue capillary glucose testing.*	**1**	5	0	96.4%	**Yes**
	**2**	5	0	91.3%	**Yes**
20. Descontinuar a colheita de estudos laboratoriais e de imagem sem impacto no conforto. *Discontinue the collection of laboratory and imaging studies without impact on comfort.*	**1**	5	0	96.4%	**Yes**
	**2**	5	0	100%	**Yes**
21. Descontinuar antibiotioterapia, aminas, profilaxias e terapêutica sem imediato impacto no conforto. *Discontinue antibiotic therapy, amines, prophylaxes, and therapies without immediate impact on comfort.*	**1**	5	0	100%	**Yes**
	**2**	5	0	100%	**Yes**
22. Rever necessidade de fluidoterapia e seu débito. *Review the need for fluid therapy and its rate.*	**1**	5	0	100%	**Yes**
	**2**	5	0	100%	**Yes**
23. Em contexto de descontrolo sintomático, considerar o contacto com a Equipa de Cuidados Paliativos. *If symptomatic decompensation occurs, consider contacting the palliative care team.*	**1**	5	0	100%	**Yes**
	**2**	5	0	95.7%	**Yes**
Intervenções a implementar ou reforçar importância *Interventions to be implemented or reinforced*					
24. Otimizar cuidados orais. *Optimize oral care.*	**1**	5	0	100%	**Yes**
	**2**	5	0	100%	**Yes**
25. Certificar da disponibilidade de utensílios para higiene e humidificação da boca. *Ensure the availability of utensils for mouth hygiene and humidification.*	**1**	5	0	96.4%	**Yes**
	**2**	5	0	95.7%	**Yes**
26. Considerar o uso de substituto de saliva. *Consider the use of a saliva substitute.*	**1**	4	2	67.8%	No
	**2**	4	2	65.2%	No
27. Otimizar cuidados à pele. *Optimize skin care.*	**1**	5	0	92.7%	**Yes**
	**2**	5	0	95.7%	**Yes**
Cuidados pós-morte *Post-mortem care*					
28. O doente é cuidado com respeito e dignidade durante a prestação dos cuidados pós-morte. *The patient is cared for with respect and dignity during post-mortem care.*	**1**	5	0	92.9%	**Yes**
	**2**	5	0	91.3%	**Yes**
29. São cumpridas as precauções universais e procedimentos relacionados com o controlo de infecção de acordo com a política da instituição. *Universal precautions and infection control procedures are followed according to the institution’s policy.*	**1**	5	1	96.4%	**Yes**
	**2**	4	1	95.7%	**Yes**
30. Satisfação das necessidades espirituais, religiosas, culturais e de rituais. *The patient’s spiritual, religious, cultural, and ritual needs are satisfied.*	**1**	5	0	92.9%	**Yes**
	**2**	5	0	91.3%	**Yes**
31. É cumprida a política da instituição relacionada com os desfibrilhadores cardíacos implantados. *The institution’s policy on implanted cardiac defibrillators is followed.*	**1**	5	0	92.9%	**Yes**
	**2**	5	0	95.7%	**Yes**
32. O serviço tem protocolos de procedimentos pós-morte e de cuidados a ter com o cadáver. *The service has protocols for post-mortem procedures and care of the deceased.*	**1**	5	0	92.9%	**Yes**
	**2**	5	0	82.6%	**Yes**
33. É cumprida a política da instituição relacionada com a guarda dos pertences/valores do doente. *The institution’s policy on custody of the patient’s belongings/valuables is followed.*	**1**	5	0	96.4%	**Yes**
	**2**	5	0	95.7%	**Yes**
34. A família é informada dos procedimentos burocráticos relacionados com o falecimento, o que pode incluir informação escrita (folheto). *The family is informed of the bureaucratic procedures related to the patient’s death, which may include written information (leaflet).*	**1**	5	0	92.9%	**Yes**
	**2**	5	0	100%	**Yes**
35. A equipa assistente (se não integrada na terapêutica atual) é informada do falecimento. *The attending team (if not part of the current therapeutic team) is informed of the patient’s death.*	**1**	5	1	89.3%	**Yes**
	**2**	5	1	91.3%	**Yes**
Revisão do diagnóstico de situação de últimas horas ou dias de vida *Review of the diagnosis of imminent death syndrome*					
36. Revisão do diagnóstico de situação de últimas horas ou dias de vida na presença de 1 dos seguintes critérios: (a) melhoria do nível de consciência, capacidade funcional, ingestão oral, mobilidade, capacidade de realizar autocuidado; (b) preocupação sobre o plano de cuidados quer seja pelo doente, parente ou elemento da equipa; (c) se passaram 3 dias desde a última avaliação completa pela equipa multidisciplinar. *The diagnosis of imminent death syndrome should be reviewed in the presence of 1 of the following criteria: (a) improvement in the patient’s level of consciousness, functional capacity, oral intake, mobility, or ability to perform self-care; (b) concern about the care plan from the patient, relative, or team member; or (c) 3 days have passed since the last comprehensive assessment conducted by the multidisciplinary team.*	**1**	5	1	100%	**Yes**
	**2**	5	0	95.7%	**Yes**
Práticas farmacológicas de situação de últimas horas ou dias de vida *Pharmacological practices in the last hours or days of life*					
37. Para o sintoma dor em regime hospitalar: Ausência de sinais de dor: Dose de resgate de morfina (2 mg EV ou 2.5 mg SC ou 1/6 da dose diária de morfina, até qh). Dor presente: Doses regular e de resgate, de acordo com tratamento proposto para dor. Não iniciar ou aumentar dose de opióides transdérmicos nesta fase. *For pain symptoms in the hospital setting: * *Absence of pain signs: Administer a rescue dose of morphine (2 mg IV or 2.5 mg SC or 1/6 of the daily morphine dose, up to qh).* *Presence of pain: Administer regular and rescue doses according to the proposed pain treatment. Do not initiate or increase the dose of transdermal opioids at this stage.*	**1**	5	1	89.2%	**Yes**
	**2**	5	1	87.0%	**Yes**
38. Para o sintoma dor no doente em regime domiciliar: Atuar da mesma forma que no regime hospitalar. Outras alternativas parentéricas: Fentanil transmucoso ou sublingual. Comprimidos de morfina de ação prolongada e de ação rápida podem ser administradas, por poucos dias, por via retal (nas doses semelhantes às orais; acautelando a sua não expulsão da ampola retal). *For pain symptoms in the patient under domiciliary care: Act in the same way as in the hospital setting. Other parenteral alternatives: Administer transmucosal or sublingual fentanyl. Prolonged-release and rapid-release morphine tablets can also be administered, for a few days, rectally (in similar doses to the oral treatment, ensuring that they are not expelled from the rectal ampoule).*	**1**	4	2	67.9%	No
	**2**	4	2	65.2%	No
39. Para o sintoma dispneia no doente em regime hospitalar: Ausência de sinais de dificuldade respiratória: Dose de resgate de morfina (2 mg EV ou 2.5 mg SC ou 1/6 da dose diária de morfina, até qh). Presença de sinais de dificuldade respiratória: Otimizar posicionamento. Corrigir hipoxia. Doses regular e de resgate, de acordo com tratamento proposto para dor. Não iniciar ou aumentar dose de opióide transdérmicos nesta fase. Se ansiedade concomitante, utilizar midazolam 2.5 mg SC ou 1–2 mg EV até qh ou lorazepam 0.5 mg sublingual até q6h. *For dyspnea symptoms in the patient in the hospital setting: * *Absence of signs of respiratory distress: Administer a rescue dose of morphine (2 mg IV or 2.5 mg SC or 1/6 of the daily morphine dose, up to qh). * *Presence of signs of respiratory distress: Optimize positioning. Correct hypoxia. Administer regular and rescue doses, according to the proposed treatment for pain. Do not initiate or increase the dose of transdermal opioids at this stage. If concurrent with anxiety, use midazolam 2.5 mg SC or 1–2 mg IV up to qh or lorazepam 0.5 mg sublingual up to q6h.*	**1**	4	1	85.7%	**Yes**
	**2**	4	1	91.3%	**Yes**
40. Para o sintoma dispneia no doente regime domiciliar: Atuar da mesma forma que no regime hospitalar. Outras alternativas parentéricas: Fentanil transmucoso ou sublingual. Comprimidos de morfina de ação prolongada e de ação rápida podem ser administradas, por poucos dias, por via retal (nas doses semelhantes ás orais; acautelando a sua não expulsão da ampola retal). O diazepam retal é alternativo ao midazolam, em doses de 10 mg até q20–30minutos. *For dyspnea symptoms in the patient under domiciliary care: Act in the same way as in the hospital setting. Other parenteral alternatives: Administer transmucosal or sublingual fentanyl. Prolonged-release and rapid-release morphine tablets can also be administered, for a few days, rectally (in similar doses to the oral treatment, ensuring that they are not expelled from the rectal ampoule). Diazepam administered rectally is an alternative to midazolam in doses of 10 mg up to q20–30 min.*	**1**	4	2	67.8%	No
	**2**	4	2	78.3%	No
41. Para o doente inquieto em regime hospitalar: Ausência de inquietação: Dose de resgate de midazolam 2.5 mg SC qh *ou* midazolam 1 mg EV qmin, até encerramento de pálpebras Presença de inquietação: Excluir desconforto físico (dor, dispneia), retenção urinária ou fecal. Otimizar posicionamento do doente. Dose inicial de 5–10 mg SC de midazolam, seguido de doses de resgate de 2.5–5 mg qh *ou* midazolam 1 mg EV qmin, até encerramento de pálpebras. *For the restless patient in the hospital setting: * *Absence of restlessness: Administer a rescue dose of midazolam 2.5 mg SC up to qh or midazolam 1 mg IV up to qmin until the patient’s eyelids close. * *Presence of restlessness: Exclude physical discomfort (pain and dyspnea) and urinary or fecal retention. Optimize patient positioning. Administer an initial dose of 5–10 mg SC of midazolam, followed by rescue doses of 2.5–5 mg up to qh or midazolam 1 mg IV up to qmin until the patient’s eyelids close.*	**1**	4	1	82.2%	**Yes**
	**2**	4	1	82.6%	**Yes**
42. Para o doente inquieto em regime domiciliar: Atuar da mesma forma que no regime hospitalar. O diazepam retal é alternativo ao midazolam, em doses de 10 mg até q20–30minutos. *For the restless patient under domiciliary care: Act in the same way as in the hospital setting. Diazepam administered rectally is an alternative to midazolam, in doses of 10 mg up to q20–30 min.*	**1**	4	1	78.9%	No
	**2**	4	1	78.5%	No
43. Para o doente com náusea/vómito do doente em regime hospitalar: Se náusea/vómitoausente: Dose de resgate de metoclopramida de 10 mg SC ou EV até q2h Presença de náusea/vómito: Otimizar cuidados orais Dose regular de metoclopramida 10 mg EV ou SC q6h e de resgate 10 mg EV ou SC até q2h *For the patient with nausea/vomiting in the hospital setting:* *Absence of nausea/vomiting: Administer a rescue dose of metoclopramide 10 mg SC or IV up to q2h.* Presence of nausea/vomiting: *Optimize oral care. Administer a regular dose of metoclopramide 10 mg IV or SC up to q6h and a rescue dose of 10 mg IV or SC up to q2h.*	**1**	4	1	78.5%	No
	**2**	4	1	78.5%	No
44. Para o doente com náusea/vómito do doente em regime domiciliar: Atuar da mesma forma que no regime hospitalar. *For the patient with nausea/vomiting under domiciliary care: Act in the same way as in the hospital setting.*	**1**	4	2	71.4%	No
	**2**	4	2	73.9%	No
45. Para o doente com estertor em regime hospitalar: Se estertor ausente: Dose de resgate de butilescopolamina 20 mg SC ou EV, até q4h. Presençaestertor: -posicionamento correto. -redução/suspensão de aporte hídrico. -butilescopolamina (20 mg q4–8h EV/SC). -aspiraçãocavidade oral (não orofaríngea). -teste com diurético (furosemida 40–60 mg SC/EV) (principalmente se insuficiência cardíaca associada). -se frequência respiratória acima de 20–25 cpm, controlar o esforço com opióide, conforme descrito para a dispneia. *For the patient with a death rattle in the hospital setting: * *Absence of a death rattle: Administer a rescue dose of butylscopolamine 20 mg SC or IV up to q4h.* *Presence of a death rattle: Correct positioning. Reduce/suspend fluid intake. Administer butylscopolamine (20 mg q4–8h IV/SC).Oral cavity aspiration (not oropharyngeal). Conduct a test with a diuretic (furosemide 40–60 mg SC/IV) (mainly if associated with heart failure). If the respiratory rate is above 20–25 cpm, control it with opioids, as described for dyspnea.*	**1**	5	1	92.8%	**Yes**
	**2**	5	1	78.3%	No
46. Para o doente com estertor em regime domiciliar: Atuar da mesma forma que no regime hospitalar O colírio de atropina 1% pode ser alternativa à butilescopolamina: 1 gota sublingual, até q4h. *For the patient with a death rattle under domiciliary care: Act in the same way as in the hospital setting. Atropine 1% eye drops can be an alternative to butylscopolamine: 1 drop sublingual, up to q4h.*	**1**	4	2	75%	No
	**2**	4	3	73.9%	No
47. Para o doente com febre em regime hospitalar: Utilizar paracetamol, AINES ou corticoesteroides de acordo com o perfil do doente, evitando invasividade e iatrogenia. *For patients with fever in the hospital setting: Use paracetamol, NSAIDs, or corticosteroids according to the patient’s profile, avoiding invasiveness and iatrogeny.*	**1**	5	1	96.4%	**Yes**
	**2**	5	1	95.7%	**Yes**
48. Para o doente com febre em regime domicilar: Atuar da mesma forma que no regime hospitalar *For patients with fever under domiciliary care: Act in the same way as in the hospital setting.*	**1**	5	1	92.8%	**Yes**
	**2**	5	1	95.7%	**Yes**

*: 1—fully disagree; 5—fully agree.**: IQR: interquartile 25–75 range.

**Table 3:** The topics covered by the comments left by the respondents on the Delphi method questionnaire and their numbers (in brackets).

**Diagnosis of end-of-life situation in the last hours or days of life** Difficulties in interpreting the concept of “ethically reversible” (3 comments). The need to consider other symptoms in addition to those presented (6 comments). Emphasis on the multidisciplinary nature of the diagnostic process, but insistence that the final decision should be made by the physician (6 comments). Difficulty in diagnosing dying in non-oncological diseases (2 comments).
**Assessment of the patient and family’s communication skills** Note that valid advance directives are not only those formally registered in RENTEV * but also those known by the team and recorded in the medical history (5 comments).
**Assessment of the physical, psychological, and spiritual needs of the patient and family** Implementing measures of comfort and privacy can be challenging “in overcrowded environments” (1 comment). The possibility of continuous accompaniment may only be partial and at the discretion of the family (1 comment).
**Surveillance and documentation of the presence of 5 cardinal symptoms** Symptom monitoring may need to be more frequent until effective control is achieved (1 comment). The instrument could include an intensity scale, not just presence/absence (1 comment). Cognitive changes can affect the patient’s report of complaints, and it may only be possible to monitor signs of pain, respiratory difficulty, or changes in mental status (1 comment).
**Interventions to implement or reinforce important procedures** Oral care should focus on mucosal hydration, regardless of the method used (5 comments).
**Pharmacological treatment of symptoms** The type of prescription for domiciliary care depends significantly on the existing resources in the community (5 comments). Concerns about the off-label use of active ingredients (2 comments). Concerns about discomfort or indignity of the proposed method of drug administration via the rectal route (2 comments). Proposals for the use of oral solutions via the buccal route (2 comments). Concerns about the fragility of patients and organ dysfunction affecting the standardization of proposed drug dosages (5 comments). Reference to the little scientific evidence for the use of antisecretory agents (butylscopolamine and atropine) (2 comments). Reference to the opportunity for preventive rather than therapeutic use of antisecretory agents (1 comment).

* RENTEV: Registo Nacional do Testamento Vital (National Advance Directives Registry).

## Data Availability

The datasets presented in this article are not readily available because as determined by the Ethics Committee, dued to confidenciality issues, the data collected will only be used for the purpose of the study and will be kept for up to 1 year after publication of the work. Requests to access the datasets should be directed to Rui Carneiro: rui.pimenta.carneiro1@hospitaldaluz.pt or ruicarneiro77@gmail.com.
